# Supplementary material for: HDSI: High dimensional selection with interactions algorithm on feature selection and testing
Source: PLoS One. 2021 Feb 16;16(2):e0246159. doi: 10.1371/journal.pone.0246159 (PMC7886179; doi:10.1371/journal.pone.0246159)
Supplement: Attachment — Submitted filename: PLoS_one_HDSI.pdf [file pone.0246159.s001.pdf]

Report on 'HDSI: High Dimensional Selection with Interactions Algorithm on Feature Selection and Testing'

August 8, 2020

The authors have proposed a new method (HDSI) for handling interaction effects in high-dimensional regression by building on the idea of random lasso but making substantial changes to the implementation. Overall, I think it is a new method that attempts to solve an important problem and illustrates its performance through multiple real data analysis and one simulation study.

In what follows, I will restrict my review to technical details and correctness and presentation.

Firstly, I think the presentation is a bit pedestrian and could have been better. In particular, when writing the algorithmic version of HDSI. Also, the motivation can be improved, the benefits of using the proposed method and the issues with random Lasso should be emphasized more in the introduction.

My comments on the paper are below, not necessarily in the order of importance.

1. Since the main contribution of this paper is proposing a new method for handling high-dimensional regression that relies on many tuning parameters, it would be useful to supply the codes for implementing the proposed method in a simulated or real data-set, either through a public repository such as github or an R package. This will also ensure reproducibility and greater use of the method by the community at large.

2. Lasso was proposed in 1996 and since then there have been many, many variants of the Lasso, including extensions for more complicated structures or non-convex penalties or more recent debiased Lasso or LAVA. It seems some discussion of the literature on sparsity is warranted. See Tibshirani [2014].

3. The main motivation for HDSI (or random lasso) is that Lasso can not select more than n features in large p, small n problems, which is resolved by methods like Elastic net. This brings a natural question, why not compare with elastic net as well, or more advanced regularization methods like de-biased Lasso?

4. Page 13: q^2 is a bad notation. Maybe make this $q^{(2)}$?

5. page 13, line 152: what is meant by 'little flexibility in coefficient estimation'? Can you elaborate or add a reference?

6. page 14, line 174: same as before? Either explain why random Lasso under-estimates or provide a reference. As a matter of fact, Lasso has a non-vanishing bias in the tails [Carvalho et al., 2009] but it's not always 'under'-estimation.

7. Algorithm 2: How do you make sure that the 2^q is less than n? It seems this poses a restriction on how many main effects and interactions can be considered in the model and the simulation study conducted has the problem set to avoid situations where the size of model with interaction terms is larger than the sample size.

8. Algorithm 2: Too many terms that are explained / defined much later in the text, e.g. L, b_j, Rsq_{ij}. Please define them before using them in an algorithm.

9. Also comment on Algo 2: Lasso-based CI's are not reliable [Chatterjee & Lahiri, 2011].

10. page 17, line 221: if you are calculating bootstrap-based CI's, why care about Normal distribution of $\hat{\beta}_j$? In fact, bootstrap works better when the statistics under consideration is either non-Gaussian or non-linear.

11. page 18, line 242: R^2 does not follow a Normal distribution, might not even be symmetric. Why use the $\mu \pm f \times \sigma$ approach? Also, how do you choose f? (Please do not use $*$ for denoting a product, if necessary use $\times$.)

12. As explained before, the simulation study is designed in a way where the number of true non-zero β's and interaction terms are very small (2-4), and it is not clear how that is against the main motivation (limitation of Lasso to select $\leq n$ coefficients).

13. Among the methods compared (e.g. Table 2), did the regression or lasso/random lasso have any interaction terms in the model at all? It seems only the group lasso had a provision for including interaction terms. If that's the case, most of the comparisons are meaningless.

14. On Table 2, n is used to denote the number of non-zero coefficients. Statisticians use n for sample size and I think even this paper has n before to denote sample size. Please consider using a different notation.

15. Finally, a minor point: the separation/dichotomy between Statistics-based and ML-based method (Lasso vs Random Forest) is artificial (at least to this reviewer). One can club them both as supervised learning methods or more generally, statistical learning.

References

CARVALHO, C. M., POLSON, N. G. & SCOTT, J. G. (2009). Handling sparsity via the horseshoe. *Journal of Machine Learning Research W&CP* **5**, 73–80.

CHATTERJEE, A. & LAHIRI, S. N. (2011). Bootstrapping lasso estimators. *Journal of the American Statistical Association* **106**, 608–625.

TIBSHIRANI, R. J. (2014). In praise of sparsity and convexity. *Past, Present, and Future of Statistical Science* , 497–505.
