# Supplementary material for: HDSI: High dimensional selection with interactions algorithm on feature selection and testing
Source: PLoS One. 2021 Feb 16;16(2):e0246159. doi: 10.1371/journal.pone.0246159 (PMC7886179; doi:10.1371/journal.pone.0246159)
Supplement: Attachment — Submitted filename: HDSI_Response_1.0_WX_RJ.docx [file pone.0246159.s002.docx]

Response to “**HDSI: High Dimensional Selection with Interactions Algorithm on Feature Selection and Testing**"

Rahi Jain^1¶^, Wei Xu^1,2¶*^

^1^Biostatistics Department, Princess Margaret Cancer Research Centre, Toronto, Ontario, Canada

^2^Dalla Lana School of Public Health, University of Toronto, Toronto, Ontario, Canada

* wei.xu@uhnresearch.ca

We would like to thank the reviewers for the valuable suggestions and comments which have significantly improved the content and presentation of the paper.

**REVIEWER #1:**

**The authors proposed a resampling-based method to facilitate feature selection and prediction for high-dimensional data. Technically, the proposed method is an ensemble method and more like a framework, which can be flexible to incorporate any machine learning methods with feature selection including lasso, adaptive lasso and elastic net. Application efficiency should be the key point for this method. However, the paper lacks extensive evaluation and the method itself needs some parameters which are hard or heuristic to determine in real application. More efforts are needed to demonstrate the effectiveness in simulations and real applications. Below are my itemised comments.**

**RESPONSE:** We appreciate the reviewer's comments. We have addressed the specific comments in the following point by point response.

**Major:
1: In table 3 and table 6, the result of random lasso is far worse than other methods including regular lasso. Random lasso uses two-step bootstrapping to get better estimation with the sacrifice of computing time. It is hard to believe that the performance of random lasso is so poor. Many issues may be reconsidered. a): In the HDSI algorithm, it generates the interaction effect after subspace sampling. That is, after the selection of q features for a bootstrapped sample, all the possible interaction terms among the q features are created. I wonder whether you conduct this procedure similarly to random lasso. In random lasso, you should also generate interaction terms after subspace sampling q features. b): The algorithm in random lasso is quite straightforward and you may directly write your own code to implement it if there is no available R package. The author of the github page you mention is not the original author of random lasso. I doubt the correctness of this github code especially the author mentioned that the code is unable to run unless modification.**

**RESPONSE:** We appreciate the reviewer for raising the question. We checked carefully at the original Random Lasso code and found that the code does not consider the intercept term. One of the potential reason is that Random LASSO focused on selecting the features and estimating their coefficients and did not explicitly estimate the intercept term, instead, it used mean corrected output. Following the reviewer’s suggestion, we developed the new code which takes into account the intercept term. The incorporation of the intercept term has significantly improved the performance of Random Lasso. Interestingly, our developed HDSI model still out-performs Random LASSO. We have added the revised results of simulation and real studies in Table 3 and Table 6 of the revised manuscript.

**2: The number of features selected in each bootstrapped sample, q, is a crucial parameter. In the simulation and real applications, the author directly fixed it at 5 or 15, which is not an appropriate way. The q must be evaluated by cross validation for each simulated data or real application.**

**RESPONSE:** We appreciate the reviewer's comment. The reviewer is correct, q is a hyperparameter which needs optimisation for each dataset and HDSI technique. We have updated the manuscript by adding the following “Hyperparameters” subsection in section “High Dimensional Selection with Interactions (HDSI)” to reflect upon the need for *q* optimisation.

“HDSI requires three hyperparameters, namely, number of features in a sample (q), coefficient estimate quantile threshold (Qi) and minimum R2 threshold (Rf). The values of these hyperparameters depend on the dataset. Hence, hyperparameter optimisation is needed for optimal performance.”

Further, real studies results have been updated in Table 4, Table 5 and Table 6 to provide the additional results based on optimal *q* rather than a prefixed *q* hyperparameter.

**3: I believe the number of bootstrapped datasets is not a crucial parameter and there is no value added to estimate the best number of bootstrapped datasets. In random forest, there will be no overfitting along with the increase of number of trees. If computing power allows, the user should choose an adequate large number of trees for the random forest to be stable. Here for HDSI, the concept is similar, and I suggest there is no need to estimate this parameter. If the authors insist to design an algorithm to estimate the appropriate number of bootstrapped datasets, at least simulation studies are needed to show that this parameter indeed has real impact to the performance**.

**RESPONSE:** We appreciate the reviewer's comment. Similar to random forest, this study uses both bootstrapping and random feature sampling. The increase in bootstraps would ensure both stable results and the occurrence of sampling of each feature. However, no information is available on the minimum number of bootstraps needed to achieve stability. The current study, similar to random LASSO, relies upon the coefficient estimate of the features in the pooling step to perform feature selection. In order to make a reliable pooled coefficient estimation, it is essential to evaluate the minimum number of coefficient estimates sample for a feature. This sample number is obtained from Lehr's Equation and is back calculated to estimate the minimum number of bootstraps. In order to clarify the viewpoint, we have changed the “Number of Bootstraps” section to “Number of Feature Samples” section. The first paragraph have also been revised as follows:

“The pooling criteria is dependent on the feature performance in different models. Accordingly, it is vital to ensure that every feature is sampled multiple times. The current Random LASSO algorithm provides no statistical guideline to determine the number of bootstrap times a feature should be selected and modeled.”

**4: For the feature selection part, authors proposed to use three criteria, quantile, confidence interval and R^2 simultaneously, which induce too many parameters which needs to be specified in real applications. The guidance and extensive evaluation should be given.**

**RESPONSE:** We appreciate the reviewer's comment. Following the reviewer’s suggestion, we have reduced the pooling criteria from three to two namely quantile based criteria (Q_i_) and R^2^ based criteria (R_f_) to avoid the issue of too many hyperparameters. Consequently, the model uses three hyperparameters namely *q*, Q_i_ and R_f_. These hyperparameters are optimised for each dataset. The following “Hyperparameters” subsection is added in section “High Dimensional Selection with Interactions (HDSI)” to reflect upon the need for hyperparameters optimisation.

“HDSI requires three hyperparameters, namely, number of features in a sample (q), coefficient estimate quantile threshold (Qi) and minimum R2 threshold (Rf). The values of these hyperparameters depend on the dataset. Hence, hyperparameter optimisation is needed for optimal performance.”

In order to showcase the need for optimisation, Scenario1 of simulation study results (Table 2 and Table 3) and all real data studies results (Table 4, Table 5 and Table 6) are updated from optimal hyperparameters rather than prefixed hyperparameters. In case of simulated data, genetic algorithm is used to select the optimal hyperparameters. In the case of real data studies, a grid search approach is used to select the optimal hyperparameters.

**Minor:
1: Line 160: boostrapped->bootstrapped
2: Please put i=1….B behind min or max in all the notations like min(Rsq_ij) or max(Rsq_ij).
3: The form between line 147 and 148 looks not very nice. Please reformat and polish this form. Also, the form between line 184 and 185.**

**Response:** Thanks for pointing these out. We have fixed these typos and formatting errors in the revised manuscript accordingly.

**REVIEWER #2:**

**The authors have proposed a new method (HDSI) for handling interaction effects in high- dimensional regression by building on the idea of random lasso but making substantial changes to the implementation. Overall, I think it is a new method that attempts to solve an important problem and illustrates its performance through multiple real data analysis and one simulation study.**

**RESPONSE:** We thank the reviewer for the encouraging comments on our manuscript.

**In what follows, I will restrict my review to technical details and correctness and presentation. Firstly, I think the presentation is a bit pedestrian and could have been better. In particular, when writing the algorithmic version of HDSI. Also, the motivation can be improved, the benefits of using the proposed method and the issues with random Lasso should be emphasised more in the introduction.**

**RESPONSE:** We appreciate the reviewer's comments. Following the reviewer’s suggestion, we have revised the “Introduction” section to clarify the motivation, the benefits of the proposed methods, and the issues with Random LASSO.

“In the case of LASSO-based techniques, they may select only the interaction feature while ignoring their marginal features. Further, random LASSO is comprehensive in feature selection and could outperform elastic net, but it is also computationally intensive due to its two-step approach (Wang et al., 2011). Random LASSO performs both feature selection and estimation but does not give the user flexibility to stop the process at feature selection or use techniques other than LASSO or adaptive LASSO. It does not provide a guideline to address the interaction terms in the model as well as the significance of estimated coefficients. Further, the estimates may suffer from systematic bias as during feature selection and estimation it assigns zero value to features not sampled. Additionally, its selection step may have interpretability issues as it uses a user assigned threshold value to determine the feature performance.”

**My comments on the paper are below, not necessarily in the order of importance.
1. Since the main contribution of this paper is proposing a new method for handling high-dimensional regression that relies on many tuning parameters, it would be useful to supply the codes for implementing the proposed method in a simulated or real dataset, either through a public repository such as github or an R package. This will also ensure reproducibility and greater use of the method by the community at large.**

**RESPONSE:** We agree with the reviewer's comments. The R code for simulation data and real dataset is added to the github and the link to the code is as follows: <https://github.com/rahijaingithub/HDSI.git>

**2.Lasso was proposed in 1996 and since then there have been many, many variants of the Lasso, including extensions for more complicated structures or non-convex penalties or more recent debiased Lasso or LAVA. It seems some discussion of the literature on sparsity is warranted. See Tibshirani [2014].**

**RESPONSE:** We appreciate the reviewer's comments. Following the reviwer’s suggestion, more literature and discussion are added on the extensions of LASSO in the 'Introduction' section.

“Besides classic LASSO, many extensions have been proposed. Elastic LASSO can be used for p>n cases, however, it may not be immune from selecting the noise variables (Zou and Hastie, 2005). Group LASSO is another approach which has been used to improve the performance of LASSO in selecting variables which need to be selected in a group such as marginal and interaction terms. However, they follow a selection hierarchy and may not select interaction terms if marginal features are not selected. Further, the groups need to be predefined (Emmert-streib and Dehmer, 2019).”

**3.The main motivation for HDSI (or random lasso) is that Lasso can not select more than n features in large p, small n problems, which is resolved by methods like Elastic net. This brings a natural question, why not compare with elastic net as well, or more advanced regularisation methods like de-biased Lasso?**

**RESPONSE:** We appreciate the reviewer for raising the question. LASSO and adaptive LASSO are used both for comparison because they are the commonly used approaches in penalised regression. Further, the focus is on showcasing the HDSI ability to improve the capability of an existing technique. This objective is achieved by comparing the technique with and without HDSI methodology. Additionally, Elastic net may solve the problem of p>n. However, (Wang et al., 2011) showed that random LASSO may perform better or at par with Elastic net. Hence, other techniques are not considered for further comparison. We agree with the reviewer that, the suggested techniques could be compared in the future research which may focus more on the robustness of HDSI.

**4.Page 13: q^2^ is a bad notation. Maybe make this q^(2)^?**

**RESPONSE:** We appreciate the reviewer's comments and the notation is updated in the revised manuscript.

**5.page 13, line 152: what is meant by ‘little flexibility in coefficient estimation’? Can you elaborate or add a reference?**

**RESPONSE:** Thanks for pointing it out. While, the issue of "little flexibility in coefficient estimation" is explained in detail in Section "Procedure Flexibility", the suggestion is accepted. Random LASSO restricts the user to use either LASSO or adaptive LASSO for estimating the variable coefficients, these techniques may not always be the most desirable approach for the user. A brief explanation is added in “High Dimensional Selection with Interactions (HDSI)” section.

“One of the limitations is that it is computationally intensive and provides little flexibility in the coefficient estimation of selected features as it allows use of only LASSO (or, Adaptive LASSO) for coefficient estimation.”

**6.page 14, line 174: same as before? Either explain why random Lasso under-estimates or provide a reference. As a matter of fact, Lasso has a non-vanishing bias in the tails [Carvalho et al., 2009] but it’s not always ‘under’-estimation.**

**RESPONSE:** We agree with the reviewer's comment and updated the manuscript. (Wang et al., 2011) had mentioned that bias is reduced but not eliminated from Random lasso. Hence, the statement in the manuscript is edited to highlight the bias issue, since a user may prefer another technique for coefficient estimation.

“Further, random LASSO may bias the coefficients (Wang et al., 2011).”

**7.Algorithm 2: How do you make sure that the 2^q^ is less than n? It seems this poses a restriction on how many main effects and interactions can be considered in the model and the simulation study conducted has the problem set to avoid situations where the size of model with interaction terms is larger than the sample size.**

**RESPONSE:** We appreciate the reviewer's comments. The current methodology segments the high dimensional feature space to low dimensional feature space to enable the application of classical statistical approaches on the high dimension feature set. Hence, the individual model is restricted by the number of main effects and interaction effects that it can accommodate. However, HDSI as a whole is not restricted, since it pools results from multiple restricted models.

**8.Algorithm 2: Too many terms that are explained / defined much later in the text, e.g. L, b_j_,R*sq*_ij_. Please define them before using them in an algorithm.**

**RESPONSE:** Thanks for pointing it out. Following the reviewer’s suggestion, we added the terms explanation in the Algorithm of the revised manuscript.

**9.Also comment on Algo 2: Lasso-based CI’s are not reliable [Chatterjee & Lahiri, 2011].**

**RESPONSE:** We appreciate the reviewer for raising the question. We agree that it is the limitation of the current study. The current methodology allows use of different statistical techniques to build models, including LASSO. We agree that LASSO results may not be consistent across the bootstraps. If LASSO is used in HDSI, HDSI will also face a similar issue. This issue would be taken up in future research. The limitation is added in the "Conclusion and Discussion" section.

“The HDSI model allows the use of different statistical techniques to build models. However, it may not be able to address all the limitations of any given technique. For instance, LASSO results may not be consistent across the bootstraps [33]. Future research could try to develop HDSI approaches which can deal with consistency limitations of techniques.”

Nevertheless, the studies by (Bach, 2008) and (Wang et al., 2011) suggest that LASSO selects relevant variables at higher frequency as compared to irrelevant variables and Bootstraps improve the selection frequency of relevant variables. In the case of pooling step for feature selection, (Bach, 2008) uses stringent criteria of non-zero coefficient in all samples. (Wang et al., 2011) uses a lenient approach of relying upon the mean coefficient value and using a heuristically defined threshold to select the features. Our approach replaces heuristically defined threshold with quantile interval to improve the interpretability of HDSI results.

**10.page 17, line 221: if you are calculating bootstrap-based CI’s, why care about Normal distribution of** ${\hat{\boldsymbol{\beta}}}_{\boldsymbol{j}}$**? In fact, bootstrap works better when the statistics under consideration is either non-Gaussian or non-linear.**

**RESPONSE:** We appreciate the reviewer's comment. We have updated the manuscript and remove the normality part. Only lower and upper quantile values are used for the pooling step. The change is made in the “Feature Selection” section.

“HDSI selects the features which fulfil two criteria (Step I(g), Algorithm 2). Firstly, the coefficient estimates between lower and upper quantile values should not contain zero. The quantile (Qi) is a hyperparameter which needs to be optimised where lower quantile is Qi/2, and upper quantile is 100-Qi/2.” (Line 239-242)

**11.page 18, line 242: R2 does not follow a Normal distribution, might not even be symmetric. Why use the µ ± f × σ approach? Also, how do you choose f ? (Please do not use ∗ for denoting a product, if necessary use ×.)**

**RESPONSE:** We appreciate the reviewer's comments. HDSI performs both sample and feature sampling, so a feature will be incorporated in multiple models. It is assumed that the models containing target features should have better goodness of fit (R^2^) as compared to models containing only noise features. Consequently, the "poor" R^2^ values should correspond to noisy features. Accordingly, a hyperparameter is defined to determine the cut-off for "poor" R^2^ value. Rather than using any threshold value, we transform the threshold value into the form (µ ± R_f_ × σ), where R_f_ is the hyperparameter which needs to be optimised, to provide better interpretation as compared to using threshold value R_f_. Following the reviewer’s suggestion, we have revised the manuscript by changing the notation * to ×.

**12.As explained before, the simulation study is designed in a way where the number of true non- zero β’s and interaction terms are very small (2-4), and it is not clear how that is against the main motivation (limitation of Lasso to select ≤ n coefficients).**

**RESPONSE:** We appreciate the reviewer's comment. In our simulation studies, the number of target variables are kept less than *n* to enable the comparison with LASSO and other classic statistical regression models.

**13.Among the methods compared (e.g. Table 2), did the regression or lasso/random lasso have any interaction terms in the model at all? It seems only the group lasso had a provision for including interaction terms. If that’s the case, most of the comparisons are meaningless.**

**RESPONSE:** We appreciate the reviewer's comment. Other than group lasso, no other method had interaction terms, since none of them inherently consider the interaction terms. The purpose of using regression/lasso/adaptive lasso is to showcase that in real-world settings with unknown features, it may be more beneficial to use techniques that consider interaction terms as compared to those which do not.

**14.On Table 2, n is used to denote the number of non-zero coefficients. Statisticians use n for sample size and I think even this paper has n before to denote sample size. Please consider using a different notation.**

**RESPONSE:** We appreciate the reviewer's comment. We have revised the manuscript accordingly to change *n* to *s* for denoting the number of non-zero coefficients.

**15.Finally, a minor point: the separation/dichotomy between Statistics-based and ML-based method (Lasso vs Random Forest) is artificial (at least to this reviewer). One can club them both as supervised learning methods or more generally, statistical learning.**
**RESPONSE:** We appreciate the reviewer's comment. We agree that both statistics and machine learning based approaches can be clubbed together into supervised/statistical learning.

**References**

Bach FR. 2008. Bolasso : Model Consistent Lasso Estimation through the BootstrapProceedings of the 25th International Conference on Machine Learning. Helsinki. pp. 33–40.

Emmert-streib F, Dehmer M. 2019. High-Dimensional LASSO-Based Computational Regression Models : Regularization, Shrinkage, and Selection. *Mach Learn Knowl Extr* **1**:359–383.

Wang BS, Nan B, Rosset S, Zhu J. 2011. Random lasso. *Ann Appl Stat* **5**:468–485.

Zou H, Hastie T. 2005. Regularization and variable selection via the elastic net. *J R Stat Soc Ser B Stat Methodol* **67**:301–320.
