# Supplementary material for: HDSI: High dimensional selection with interactions algorithm on feature selection and testing
Source: PLoS One. 2021 Feb 16;16(2):e0246159. doi: 10.1371/journal.pone.0246159 (PMC7886179; doi:10.1371/journal.pone.0246159)
Supplement: Attachment — Submitted filename: HDSI_Response_2.0_WX_RJ.docx [file pone.0246159.s003.docx]

Response to “**HDSI: High Dimensional Selection with Interactions Algorithm on Feature Selection and Testing**”

Rahi Jain^1^, Wei Xu^1,2*^

^1^Biostatistics Department, Princess Margaret Cancer Research Centre, Toronto, Ontario, Canada

^2^Dalla Lana School of Public Health, University of Toronto, Toronto, Ontario, Canada

* wei.xu@uhnresearch.ca

We would like to thank the reviewers for the valuable suggestions and comments which have significantly improved the content and presentation of the paper.

**REVIEWER #1:**

**1: In section “Hyperparameters”, authors only mentioned the need of optimizing the hyperparameters without a practical guidance. There are three parameters needed to be determined: $q$, $Q_i$ and $R_f$, Line 336-340 mentions the hyperparameters are obtained by five-fold CV and heuristic optimization. Could authors state clearly what heuristic procedure they did? In real data (Line 416), authors mention the three parameters are selected by grid search. I am thinking an exhaustive cross validation of the combination of the three parameters seems overwhelming. I suggest authors can clearly state how they select three parameters in “Hyperparameters” Section.**

**RESPONSE:** We appreciate the reviewer’s suggestion. Following the reviewer’s suggestion, we have explained in more detail the process of selecting hyperparameters in the “Hyperparameters” section, Page 14 in the revised manuscript.

“Hyperparameter optimization is done in three steps. In first step, $Q_{i}$ and $R_{f}$ are kept constant at value five and value zero respectively and $q$ is optimized. Randomly, multiple values are generated for $q$ and value with the best predictive performance is selected. In second step, $q$ and $R_{f}$ are kept constant at the best value obtained from first step and value zero respectively, while $Q_{i}$ is optimized. Randomly, multiple values are generated for $Q_{i}$ and value with the best predictive performance is selected. In third step, $q$ and $Q_{i}$ are kept constant at the best value obtained from step one and two respectively, while $R_{f}$ is optimized. Randomly, multiple values are generated for $R_{f}$ and value with the best predictive performance is selected.”

**2: Following 1, I suggest authors do some sensitivity analysis to the hyperparameters, giving a practical region of each hyperparameter (i.e. $Q_i$ between 0.05-0.2 works well). With this, users can quickly pick a suitable parameter sets to explore. Also, it helps to reduce the computational load if users want to do cross validation over combinations of three hyperparameters (the number of combinations can be largely reduced).**

**RESPONSE:** We appreciate the reviewer’s suggestion. We performed additional simulations and added a new sub-Section “Hyperparameter Optimal Range” on Page 16, Line 331 in the revised manuscript to provide a practical region for each hyperparameter to explore for optimal values. We identified the region using Adaptive Lasso based HDSI technique and validated the region using regression based HDSI on the same simulated dataset. Further, as the identified hyperparameters values may not be globally optimal, results suggest that HDSI could provide robust performance even in non-optimal settings.

**3: Authors should spend more time to proofread. For example, line 39, p and n should use mathematical format: $p$ and $n$. Line 228: Min_RSq_j is not professional. Line 395, the space is too large and looks strange. There are many flaws like this and authors should proofread carefully and make the writing more professional.**

**RESPONSE:** Thanks for pointing these out. We have fixed these typos and formatting errors in the revised manuscript accordingly.

**4: Following 3, I feel that many equations can fit into the text. There are 16 equations numbered and most of them are not the key part of this manuscript and it is better to fit them into the text.**

**RESPONSE:** We appreciate the reviewer’s comments and reduced the number of equations from 16 to 8 in the revised manuscript.

**5: For number of bootstrapped datasets ($B$), my suggestion is to do a sensitivity analysis using simulation (i.e. B=20, 50, 100, 200, 500, 1000, 2000). At the beginning (B=20), the result will be unstable, but at certain point, the result will be stable (for example, B=500). This sensitivity analysis will be in complementary with the rule of thumb authors proposed.**

**RESPONSE:** We appreciate the reviewer’s suggestion. Following the reviewer’s suggestion, we have added a new sub-Section “Bootstraps Analysis” on Page 16, Line 349 in the revised manuscript to describe the relevance of the number of bootstraps in the model. It is observed that an increase in bootstraps improved the HDSI performance even when optimal hyperparameters values are not used. The suggested effect size 0.8 seems to provide stable results.

**REVIEWER #2:**

**I think almost all the comments that I made on the earlier version were addressed in the current draft. I am still a little bothered by the fact that the current method is essentially bounded by limitation of Lasso of selecting p <= n variables inside the HDSI algorithm. It seems using other variable selection methods might bypass this limitation. I have just a couple of minor suggestions.**

**RESPONSE:** We thank the reviewer for the comments on our manuscript. We agree that our current method does not change the inherent functionality of the existing methods. Instead, it changes the ecosystem in which these standard methods operate. This change in an ecosystem allows feature selection in high dimensional settings. We have also added this explanation in the “Conclusion and Discussion” section on Page 23, Line 472 in the revised manuscript.

**a) Include the answer to Q. 7 in the main manuscript, that is, the segment: “The current methodology segments the high dimensional feature space to low dimensional feature space to enable the application of classical statistical approaches on the high dimension feature set. Hence, the individual model is restricted by the number of main effects and interaction effects that it can accommodate. However, HDSI as a whole is not restricted, since it pools results from multiple restricted models.”**

**RESPONSE:** We appreciate the reviewer’s comment and the text is added in the “Conclusion and Discussion” section on Page 23, Line 465.

**b) On HDSI algorithm, change “feature technique” to “feature selection technique”.**

**RESPONSE:** We appreciate the reviewer’s comment and the text is changed in the Algorithm.
